# Palliative external beam radiotherapy for the treatment of tumor bleeding in inoperable advanced gastric cancer

**DOI:** 10.1186/s12885-017-3508-x

**Published:** 2017-08-12

**Authors:** Yun Hee Lee, Jeong Won Lee, Hong Seok Jang

**Affiliations:** Seoul St. Mary’s Hospital, Seoul, Republic of Korea

**Keywords:** Gastric cancer, Radiotherapy, Palliative treatment, Bleeding

## Abstract

**Background:**

To assess the outcomes and prognostic factors associated with palliative external beam radiotherapy (EBRT), administered to patients with advanced gastric cancer.

**Methods:**

Forty-two patients with bleeding gastric tumors that received EBRT for palliation were analyzed. The response to EBRT was assessed by the palliation of tumor bleeding. Patients were classified as either responders, or non-responders to EBRT. The prognostic utility of clinical and dosimetric variables was examined in a multivariate logistic regression model. The optimal dose cutoff to classify the two groups was determined with receiver operating characteristic analysis.

**Results:**

The palliation of gastric tumor bleeding after EBRT was achieved in 29 patients (69.0%). The time to resolve tumor bleeding ranged from 1 to 84 days (median, 15 days). The median duration of palliation was 14.9 weeks. The median EBRT dose was 40 Gy in responders vs. 21 Gy in non-responders, with the difference being significant (*p* < 0.001). The biologically effective dose (using α/β = 10, BED_10_) for responders was significantly higher than the BED_10_ for non-responders (median 48 Gy vs. 26.4 Gy, *p* < 0.001), and the optimal cut off value to separate the two groups was 36 Gy (*p* < 0.001). The absence of distant metastasis and the use of concurrent chemotherapy generally showed a better EBRT response (*p* = 0.079 and *p* = 0.079, respectively). In the multivariate analysis, BED_10_ ≥ 36 Gy was the most significant factor associated with EBRT response (*p* = 0.001). Overall survival (OS) and re-bleeding-free survival was median 12.6 weeks and 14.9 weeks. The responders to EBRT showed superior OS (16.6 vs. 5.1 months, *p* < 0.001). Neither acute nor chronic toxicities of grade 3 or higher were observed.

**Conclusions:**

EBRT is an effective method for treating tumor bleeding in advanced gastric cancer, and does not induce severe toxicity.

## Background

Gastric cancer is one of the most prevalent malignancies, and one of the major causes of cancer death in Korea and worldwide [1, 2]. The preferred treatment of gastric cancer for curative-intent is surgical resection. However, several patients presenting with unresectable disease are treated with chemotherapy [3]. Despite advancements in the early detection and treatment of gastric cancer, the prognosis from curative treatment remains poor, and more than 50% of the patients will develop metastatic disease [3–5]. Local symptoms that occur frequently are pain, gastric outlet obstruction, especially tumor bleeding from gastric lesions such as hematemesis, melena, and repeated anemia [6–9]. For such patients, physicians may consider several treatment options, such as endoscopic intervention, palliative gastrectomy or surgical bypass, palliative chemotherapy, or radiotherapy (RT) [10].

Of the aforementioned options, palliative external beam radiotherapy (EBRT) may be a non-invasive, safe, and readily available procedure, with relatively few restrictions regarding the eligibility for treatment [11]. Furthermore, EBRT is not only an effective procedure for the immediate reduction of symptoms, but also as a therapeutic approach that acts directly on the tumor cells.

At present, a limited number of studies for EBRT in patients with inoperable advanced gastric cancer with tumor bleeding have been reported [7, 9, 12, 13]. The aim of the present study was to evaluate the clinical outcomes after EBRT with regard to resolving tumor bleeding due to advanced gastric cancer.

## Methods

### Patients

The current study retrospectively reviewed the medical data of 42 patients with gastric cancer who experienced tumor bleeding with or without obstruction caused by the tumor, with subsequent EBRT treatment with a palliative aim. All patients were treated at Seoul St. Mary’s Hospital, Seoul, Korea between January 1991 and March 2014. The eligibility criteria included locally advanced or metastatic gastric cancer, clinical findings that indicated gastrointestinal bleeding due to gastric cancer, or laboratory findings of anemia, and no prior RT to the abdomen. Patients who had received previous management for gastric tumor bleeding were included in the present study. This study was approved by the Institutional Review Board of Catholic University of Korea (IRB number: KC14RISI0279).

### Radiotherapy

EBRT was delivered with either 6 or 10 MV photons. RT treatment planning was conducted using Computed Tomography (CT) for 27 patients (64.3%). Stomach movements were observed under fluoroscopy, and the margins were modified if necessary. Radiation was delivered to portions of the stomach in 29 patients and to the whole stomach in 13 patients. Thirty patients (71.4%) were treated with an opposed pair of anterior-posterior fields, with an additional lateral field, and 12 patients (28.6%) were treated with an opposed pair of anterior-posterior fields. Radiation field sizes ranged from 8 × 8 cm^2^ to 23 × 21 cm^2^ (median, 13 × 10 cm^2^). Radiotherapy was delivered with a median total dose of 39.6 Gy (range, 14–50.4 Gy) in a median number of 20 fractions (range, 7–28 fractions). The median duration of RT was 27 days (range, 6–69 days).

### Evaluation

The response to EBRT was assessed according to both the palliation of gastric tumor bleeding and whether a further form of management was required to control the bleeding. Re-bleeding was defined as the bleeding symptoms such as hematemesis or melena developing a second time. Overall survival (OS) was defined as the time from the start of EBRT to death from any cause. Tumor bleeding-free survival was defined as the time from the initiation of EBRT to re-bleeding caused by the gastric tumor, or death of the patients. Toxicities were graded according to the National Cancer Institute Common Toxicity Criteria version 3.0. Grade 3 or higher nausea, vomiting, asthenia, dysphagia, or epigastric pain, were considered significant toxic effects.

All survival rates were estimated by Kaplan-Meier analysis and compared by a log-rank test. A Chi-square or Fisher exact test was used to evaluate the significance of any correlation between the categorical variables and tumor response to EBRT. Mann-Whitney U tests were used to compare continuous variables between RT responders and RT non-responders. Statistically significant factors in the univariate analysis (*p*-value <0.10) and expected clinically significant factors were included in the multivariate logistic regression model. Biologically effective dose using α/β = 10 (BED_10_) for early responding tissues was computed according to the linear–quadratic model [13]. A receiver operator characteristic (ROC) curve was generated to identify the optimum dose cut-off value, with the optimal value defined as that which had the highest sensitivity and specificity. All statistical analyses were 2-sided, and a *p*-value ≤0.05 was considered statistically significant.

## Results

The patient demographics and response to RT are shown in Table 1. Seven patients were treated with concurrent chemoradiotherapy, using a chemotherapy regimen consisting of 5-fluorouracil/leucovorin. Chemotherapy was delivered concurrently with RT until 2001, with RT-alone being utilized from 2002 onward. Gastric tumor bleeding resolved with EBRT treatment in 29 of 42 patients (69.0%). The median time to the palliation of tumor bleeding from the initiation of EBRT was 15 days (range, 1–84 days). The bleeding-free duration after EBRT ranged from 2.3 to 61.0 weeks (median, 14.9 weeks). Upon univariate analysis, age, gender, performance status, pathology and differentiation, Bormann type, tumor bleeding site, history of previous treatment, or previous bleeding management were not found to be associated with bleeding control. RT parameters such as 2-dimensional or 3-dimensional technique, and RT field were not significantly associated with the tumor response to EBRT. The absence of distant metastasis and the use of concurrent chemotherapy showed better response to RT (*p* = 0.079 and *p* = 0.079, respectively). The median RT dose was 40 Gy in responders vs. 21 Gy in non-responders, with the difference being significant (*p* < 0.001). BED_10_ for responders was significantly higher than the BED_10_ for non-responders (median 48 Gy vs. 26.4 Gy, *p* < 0.001). To determine the cut-off values of BED_10_, a ROC curve was generated. The optimal cut-off value was found to be 36 Gy, with an area under curve (AUC) value of 0.901 (95% confidence interval [CI] = 0.811–0.990, *p* < 0.001). In addition, BED_10_ ≥ 36 Gy was significantly associated with bleeding control in the univariate analysis (*p* < 0.001). Distant metastasis, concurrent chemoradiotherapy, BED_10_ ≥ 36 Gy, and previous bleeding management were included in the multivariate logistic regression analysis. BED_10_ ≥ 36 Gy was the only factor associated with an improvement in gastric tumor bleeding (OR 1.142, 95% CI 1.059–1.232, *p* = 0.001).

**Table 1:** Patient demographics and response to radiotherapy

	All patients	Responder (*n* = 29)	Non-responder (*n* = 13)	*P* value
Age (years)				
≥ 65	21 (50%)	14 (48.3%)	7 (53.8%)	0.739
< 65	21 (50%)	15 (51.7%)	6 (46.2%)	
Gender				0.187
Male	26 (61.9%)	20 (69%)	6 (46.2%)	
Female	16 (38.1%)	9 (31%)	7 (53.8%)	
Clinical metastatic stage				0.079
cM0	7 (16.7%)	7 (24.1%)	0 (0%)	
cM1	35 (83.3%)	22 (75.9%)	13 (100%)	
ECOG performance status				0.217
1	11 (26.2%)	10 (34.5%)	1 (7.7%)	
2	23 (54.8%)	14 (48.3%)	9 (69.2%)	
3	8 (19%)	5 (17.2%)	3 (23.1%)	
Pathology				0.695
Adenocarcinoma	33 (78.6%)	22 (75.9%)	11 (84.6%)	
Others	9 (21.4%)	7 (24.1%)	2 (15.4%)	
Differentiation				1.0
Well differentiated	1 (2.4%)	1 (3.4%)	0 (0%)	
Moderately differentiated	17 (40.5%)	12 (41.4%)	5 (38.5%)	
Poorly differentiated	18 (42.9%)	11 (37.9%)	7 (53.8%)	
unknown	6 (14.3%)	5 (17.2%)	1 (7.7%)	
Borrmann type				0.357
1	1 (2.4%)	1 (3.4%)	0 (0%)	
2	2 (4.8%)	2 (6.9%)	0 (0%)	
3	34 (81%)	23 (79.3%)	11 (84.6%)	
unknown	6 (14.3%)	3 (10.3%)	2 (15.4%)	
Site				0.381
Upper	10 (23.8%)	9 (31%)	1 (7.7%)	
Mid	19 (45.2%)	11 (37.9%)	8 (61.5%)	
Lower	13 (31%)	9 (31%)	4 (30.8%)	
Previous Treatment				0.626
Surgery	1 (2.4%)	0 (0%)	1 (7.7%)	
Chemotherapy	29 (69%)	22 (75.9%)	7 (53.8%)	
Surgery +Chemotherapy	2 (4.8%)	1 (3.4%)	1 (7.7%)	
none	10 (23.8%)		4 (30.8%)	
Previous bleeding management				0.398
No	34 (81%)	22 (75.9%)	12 (92.3%)	
Yes	8 (19%)	7 (24.1%)	1 (7.7%)	
Concurrent chemoradiotherapy				0.079
No	35 (83.3%)	22 (75.9%)	13 (100%)	
Yes	7 (16.7%)	7 (24.1%)	0 (0%)	
Radiotherapy technique				1.000
2-dimensional	15 (35.7%)	10 (34.5%)	5 (38.5%)	
3-dimensional	27 (64.3%)	19 (65.5%)	8 (61.5%)	
Radiotherapy filed				0.495
Whole stomach	13 (31%)	8 (27.6%)	5 (38.5%)	
Partial stomach	29 (69%)	21 (72.4)%	8 (61.5%)	
Total radiation dose (Gy)	39.6 (14–50)	40 (18–50)	21 (14–40)	<0.001
Total radiation dose BED_10_^a^ (Gy)	46.7 (16.8–60)	48 (21.6–60)	26.4 (16.8–46.7)	<0.001

*Abbreviations*: *ECOG* eastern cooperative oncology group, *RT* radiotherapy, *BED* biologically effective dose

^a^Biologically effective dose when α/β ratio is presumed to be 10 for early responding tissue

**Fig. 1:**
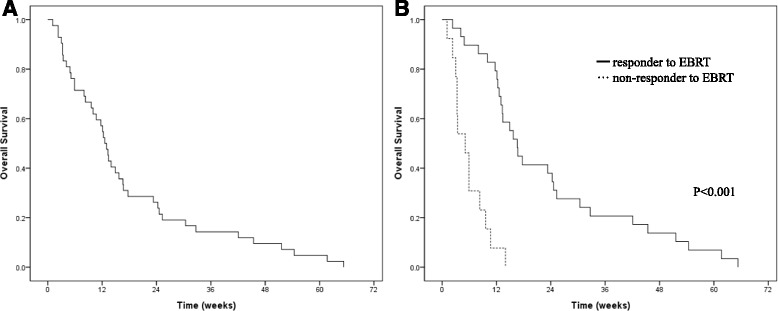
Overall survival of all patients (**a**) and based on the response to radiotherapy (**b**)

**Fig. 2:**
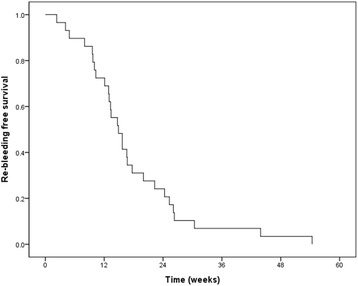
Re-bleeding-free survival of responders (*n* = 29)

The median OS was 12.6 weeks (95% CI = 10.8–14.4 weeks) (Fig. 1a). There was a significant difference in OS according to the bleeding palliation (*p* < 0.001, log-rank test) (Fig. 1b). Median OS for the responders to EBRT was 16.6 weeks (13.4–19.8 weeks), and 5.1 weeks (2.8–7.4 weeks) for non-responders. Among the 29 responders, re-bleeding occurred in 11 patients following the resolution of the hemorrhagic symptoms. The median re-bleeding-free survival was 14.9 weeks (95% CI = 11.87–17.93 weeks), as shown Fig. 2. None of the patients received additional EBRT. The patients who received a second form of hemostatic management prior to EBRT did not experience re-bleeding (*p* = 0.026).

Most acute toxicities such as nausea, vomiting, asthenia or epigastric pain caused by EBRT, were manageable. There were no grade 3 or higher toxicities in the present study.

## Discussion

The current study demonstrated the efficacy of EBRT with respect to bleeding caused by gastric cancer. Radiation dose (BED_10_ ≥ 36 Gy) was significantly associated with a bleeding cessation. The patients who underwent previous treatment with a different method for the management of tumor bleeding did not experience re-bleeding. In addition, the responders to EBRT displayed better OS compared with non-responders.

EBRT is an effective treatment option for relief of symptoms, such as pain, dysphagia, dyspnea, and tumor bleeding [14–17]. There are a limited number of studies focusing on palliative EBRT to relieve tumor bleeding in advanced gastric cancer [7, 9, 12, 13, 18, 19] (Table 2). The aforementioned studies have reported bleeding control rates of 50% to 91%, with 69% of the patients in the current study responding to EBRT.

**Table 2:** Studies involving the use of palliative external beam radiotherapy for tumor bleeding in advanced gastric cancer

Study	No	Prescribed dose	Symptom response rate	Relation RT dose and treatment outcome			Association between concurrent chemoradiotherapy and treatment outcome
				Significant dose (BED^a^)	Symptom response	Re-bleeding	
Hashimoto et al. [10]	19	40 Gy/16fx (range,2–50 Gy/1.8–3 Gy per fx)	68.4%	50 Gy_10_	Yes (*P* = 0.040)	NR ^b^	NR ^b^
Asakura et al. [7]	30	30 Gy/10 fx (90% of patients)	73%	39 Gy_10_	NR ^b^	NR ^b^	Decreased re-bleeding event (60% vs. 17.5%, *P* = 0.001)
Lee et al. [17]	23	30 Gy/10fx (range, 30-44Gy/10-22fx)	91%	NR ^b^	NR ^b^	NR ^b^	NR ^b, g^
Kim et al. [11]	20 ^c^	35 Gy/14 fx ^c^ (m/c)	70% ^c^	41 Gy_10_^d^	NR ^d^	Yes ^d^ (*P* = 0.05)	A trend towards better OS ^d^ (6- month OS 50% vs. 23%, *P* = 0.08)
Tey et al. [8]	103 ^e^	30 Gy/10fx (m/c) 8-Gy/1fx-40Gy/16fx	80.6% ^e^	39 Gy_10_^e, f^	No ^e^ (*P* = 0.78)	No ^e^ (*P* = 0.78)	NR ^b, g^
Chaw et al. [16]	44	8 Gy/fx (75%) or 20 Gy/5fx (25%)	50%	14.4 or 28 Gy_10_	No (*P* = 0.202)	NR ^b^	NR ^b, g^
Present study	42	39.6 Gy/20fx (range, 14–50.4 Gy/7-28fx)	69%	36 Gy_10_	Yes (*P* < 0.001)	No (*P* = 0.238)	A trend towards better symptom response rate (24.1% vs. 0%, *P* = 0.079)

^a^ *BED* biologically effective dose

^b^ *NR* not reported

^c^Only bleeding case

^d^Not only bleeding case

^e^Only bleeding case

^f^There was no statistical difference in response rates between low (≤39 Gy_10_) and high (>39 Gy_10_) BED

^g^No patient received concurrent chemotherapy in combination with radiotherapy

RT dose, in the form of BED_10_, was the significant factor associated with response to EBRT in the current study (*p* < 0.001). However, previous studies have reported conflicting outcomes regarding the RT dose and symptom relief. Several studies suggested the use of short-course RT because bleeding control was not affected by the total RT dose [9, 18]. By contrast, Hashimoto et al. [12], Kim et al. [13], and the current study demonstrated that higher RT dose improved the outcomes of patients. The present study attempted to ascertain the necessary RT dose using ROC analysis, with the optimal cut-off value found to be 36 Gy (BED_10_). However, the present study was unable to determine an association between the optimal cutoff value and the occurrence of re-bleeding (*p* = 0.238).

In the current study, re-bleeding did not occur in patients who received a previous form of management for tumor bleeding before EBRT treatment (*p* = 0.026). The reasons for this may be a result of tumor bleeding focus to respond easily or remained effect of the prior hemostatic management.

The role of concurrent chemotherapy to control bleeding has not been determined. Asakura et al. [7] reported that concurrent chemotherapy decreased the number of re-bleeding events, and Kim et al. [13] reported the use of concurrent chemotherapy usually resulted in an improved OS. The current study displayed a trend indicating better symptom response rates (100.0% vs. 62.9%, *p* = 0.079). All patients that received concurrent chemoradiotherapy demonstrated a response to EBRT.

Several studies have used the ratio of the period of symptom relief to the patient’s remaining life multiplied by 100 for evaluating the efficacy of EBRT for the palliation of symptoms caused by tumors [9, 13, 15, 16]. The aforementioned index concerning the use of EBRT for bleeding control was reported to have a median of 70% by Kim et al. [13], and a mean of 92.5% by Tey el al. [9]. In the present study, this index ranged from 30% to 100% (median, 100%). Tumor bleeding could induce malnutrition and dehydration, which can impede proper cancer treatment. Having a higher index may result in an improvement of patients’ quality of life [11].

There are certain limitations with respect to the inherent biases associated with a retrospective study design. There was uncertainty in assessing subjective toxicity that may result in an underestimation of toxicity. EBRT treatment was interrupted for 13 patients; 5 patients (17.2%) with response to RT and 11 (84.6%) with non-response to RT. The most common cause of interruption was a poor general condition. There is a possibility of selection bias that patients with a better performance status were prescribed higher doses of radiation resulting in a better response to RT. Although there was a small sample size in the present study, the number of patients was comparable to published studies [7, 12, 13, 18, 19].

## Conclusion

In conclusion, EBRT is a feasible and effective treatment modality for the palliation of tumor bleeding in patients with advanced gastric cancer. The current study demonstrated that a BED_10_ ≥ 36 Gy may result in an improved response to EBRT in the treatment of gastric tumor bleeding.

## Abbreviations

AUC: Area under curve
BED_10_: Biologically effective dose using α/β = 10
CCRT: Concurrent chemoradiotherapy
EBRT: External beam radiotherapy
ECOG: Eastern cooperative oncology group
OS: Overall survival
ROC: Receiver operator characteristic
RT: Radiotherapy
