# Correction to: Palliative external beam radiotherapy for the treatment of tumor bleeding in inoperable advanced gastric cancer

**DOI:** 10.1186/s12885-018-4088-0

**Published:** 2018-02-27

**Authors:** Yun Hee Lee, Jeong Won Lee, Hong Seok Jang

**Affiliations:** 1 Department of Radiation Oncology, Gyeongsang National University School of Medicine and Gyeongsang National University Hospital, Jinju, South Korea; 2 Institute of Health Sciences, Gyeongsang National University, Jinju, South Korea; 3 Department of Radiation Oncology, Kyungpook National University Medical Center, Daegu, South Korea; 4 Department of Radiation Oncology, Seoul St. Mary’s Hospital, College of Medicine, The Catholic University of Korea, Seoul, South Korea

## Correction to: BMC Cancer (2017) 17:541 DOI: 10.1186/s12885-017-3508-x

In the original version of this article [1], published on 12 August 2017, the affiliations and author contributor details were not correct. In this Correction the incorrect affiliations and author contributor details and correct affiliations and author contributor details are shown.

Originally the author affiliations and author contributor details had been published as:

Yun Hee Lee, Jeong Won Lee and Hong Seok Jang*^1^.

* Correspondence: Hong Seok Jang (hsjang11@catholic.ac.kr).

^1^Seoul St. Mary’s Hospital, Seoul, Republic of Korea.

The correct author affiliations and author contributor details are:

Yun Hee Lee, MD^1,2†^, Jeong Won Lee, MD^3†^, Hong Seok Jang, MD^4*^.

* Correspondence: Hong Seok Jang (hsjang11@catholic.ac.kr).

† Equal contributors.

^1^Department of Radiation Oncology, Gyeongsang National University School of Medicine and Gyeongsang National University Hospital, Jinju, Korea.

^2^Institute of Health Sciences, Gyeongsang National University, Jinju, Korea.

^3^Department of Radiation Oncology, Kyungpook National University Medical Center, Daegu, Korea.

^4^Department of Radiation Oncology, Seoul St. Mary’s Hospital, College of Medicine, The Catholic University of Korea, Seoul, Korea.
